# Phenocoll Hydrochlerate

**Published:** 1892-08-27

**Authors:** 


### PHENOCOLL HYDROCHLORATE.

We have in a former issue described the nature and properties of phenocoll. It has been made the subject of further investigations, and the following summary of experiments by Dr. Isaac Ott, published in his notes on "New Remedies," will be of interest. Upon frogs it produces a general paralysis, due to an action upon the cerebro-spinal axis. In rabbits it reduces the force and frequency of the heart. It kills through an action upon the respiratory centre. It diminishes vascular tension. Upon the temperature its action is one of reduction. Its effect upon the temperature, however, seems to be of short duration.*

But the drug has been used extensively in practice, and it will be no less interesting to note the results obtained by other investigators of this new synthetic remedy.

Thus Dr. Herzog has tried it in seventeen cases, viz., four cases of pulmonary phthisis, one of pleuritic effusion, one of pneumonia, abscess of liver one case, erysipelas, sciatica, in otitis media in two cases, cholelithiasis, chronic rheumatism, and in pain in a hysterical subject. In the greater number the drug produced no disagreeable after effects or secondary phenomena. Its antipyretic action manifests itself after the administration of one gramme, provided one takes care to give it at the moment the fever attains its culminating point, the temperature then begins to fall one hour after the administration, and reaches its lowest point in three to five hours; it begins to rise afresh after seven hours, but rarely later. But, given during the rise of temperature its action is less pronounced, even in doses of one gramme oft repeated. The fall of temperature is almost always accompanied by more or less profuse perspiration; each time in the case of the two phthisical patients one was able to control the night sweats by the administration of ·005 gr. with ·001 atropine, some patients presenting a trembling more or less intense during the subsequent rise of temperature; but never was a rigor observed. Nausea and vomiting in the case of one patient. In the two cases of sciatica and in one phthisical case, recourse was had to subcutaneous injections. In these the temperature fell without bad secondary results.

As an analgesic and anti-rheumatic, hydrochlorate of phenocoll was shown to be very efficacious in two cases of sciatica, decrease of the pain in one case of chronic rheumatism, and amelioration in two cases of intense cephalalgia. The author concludes that in the above-mentioned conditions the remedy may be regarded as an efficient and certain antipyretic and one free from danger; it should be given with great caution to very feeble patients, and is a good antineuralgic and anti-rheumatic remedy.†

* *Ther. Gazette*, October 15, 1891.

† *Cntrbl. f. d. Gsumte Ther.*, 1891, p. 540.

It has been administered in malarial fever, Albertone reports results in thirty-four cases which were treated with it. (*Ref. Med.* Jan. 5th, 1892.) Of these, twenty-four were permanently cured, in five the result was nil, in the remaining five it was doubtful. In some of the cases which were cured the disease was very severe, and quinine had done little or no good. The dose of phenocoll used was always one gramme, given in powder or solution, from five to seven hours before the attack. The substance produced no bad after effect, and the taste is easily masked by sugar. The attack was cut short after one or at most two doses, but his administration of the drug was continued for some days after the cessation of the fever in order to prevent relapse.*

One of the latest reports has been furnished by Cohnheim.† It has been said by that observer that phenocoll retains the antipyretic action and non-poisonous property of phenacetin; it lends itself on account of its ready solubility to quicker absorption and prompter action; moreover, it may be used by subcutaneous injection, and appears to possess the sedative effect of phenacetin in a larger degree. It was administered by Cohnheim in aqueous solution in doses of seven grains. Smaller quantities were found useful in fever of the hectic type, and average of over 3° F. fall being produced by seven grains. The time taken by the temperature to fall to the minimum varied from three to four hours; there was copious sweating, and in the rebound of the temperature slight shivering. The urine appeared dark brown, giving a reaction with perchloride of iron, but contained neither albumen nor bile.

* *Brit. Med. Jour.*, April 2, 1891.

† *Therap. Journal*, No. 1, 1892; *Pract.* Feb. 1892.

